# The Impact of Aging Policy on Societal Age Stereotypes and Ageism

**DOI:** 10.1093/geront/gnab151

**Published:** 2021-10-12

**Authors:** Reuben Ng, Ting Yu Joanne Chow, Wenshu Yang

**Affiliations:** Lee Kuan Yew School of Public Policy, National University of Singapore, Singapore, Singapore; Lloyd’s Register Institute for the Public Understanding of Risk, National University of Singapore, Singapore, Singapore; Lee Kuan Yew School of Public Policy, National University of Singapore, Singapore, Singapore; Lloyd’s Register Institute for the Public Understanding of Risk, National University of Singapore, Singapore, Singapore

**Keywords:** Medicalization of aging, Moderated mediation, Old-age support ratio, Policy agenda setting, Text as data

## Abstract

**Background and Objectives:**

While studies have researched ageism in public policy, few investigated the impact of aging policy on ageism—typically, an unintended consequence. Ageism is linked to $63 billion in health care costs, so its antecedents are of interest. We test the association between Aging Policy Agenda Setting and Societal Age Stereotypes and hypothesize a mediating pathway via Medicalization of Aging, moderated by demographics.

**Research Design and Methods:**

Scholars identified Singapore’s Pioneer Generation Policy (PGP) as one of the largest policy implementations in recent years, where the agenda was set by the Prime Minister at an equivalent State of the Union address in 2013, and US$7 billion allocated to fund outpatient health care costs for aged 65 years or older. More than 400,000 older adults received a PGP card and home visits by trained volunteers who co-devised a personalized utilization plan. We leveraged a 10-billion-word data set with more than 30 million newspaper and magazine articles to dynamically track Societal Age Stereotype scores over 8 years from pre- to postpolicy implementation.

**Results:**

Societal Age Stereotypes followed a quadratic trend: Prior to the Aging Policy Agenda Setting from 2010 to 2014, stereotypes were trending positive; after 2014, it trended downward to become more negative. Medicalization of Aging mediated the relationship between Aging Policy Agenda Setting and Societal Age Stereotypes. Furthermore, Old-age Support Ratio moderated the mediational model, suggesting that the impact of policy on medicalization is stronger when a society is more aged.

**Discussion and Implications:**

We provided a framework for policymakers to ameliorate the unintended consequences of aging policies on societal ageism—if unaddressed, it will exert an insidious toll on older adults, even if initial policies are well-intentioned.

The insidious consequences of ageism at the individual and societal levels prompted 194 member countries to collectively work through the World Health Organization on a global campaign to combat ageism (Officer & de la Fuente-Núñez, 2018). This remarkable global consensus is an awakening to ageism’s consequences on population health and its alarming prevalence in health policy (Lloyd-Sherlock et al., 2016). Ageism is defined as the discrimination of older adults based on their age (Butler, 1969) and expanded by scholars to include negative portrayals of older adults as derived from age-based stereotypes centered on illness, irrelevance, and incompetence (North & Fiske, 2015)—categorized broadly as age stereotypes. The battle against ageism is complex and evolving, with significant federal legislations like the Age Discrimination in Employment Act, first introduced in 1967, that paved the way for the abolishment of the mandatory retirement of older adults in 1986, heralding the reframing of aging as a productive life stage (Binstock, 2005). While scholars have highlighted that the modern definition of ageism may be expanded to include younger people on the other side of the age spectrum—given that youth may be more likely to suffer age discrimination in a country with stronger structural support for older people—our study focuses on ageism against older adults.

While many studies have researched ageism in public policy, few have investigated the impact of public policy on ageism. Specifically, we are concerned with the unintended impact of aging policies that aim to provide some form of financial or medical aid for older adults, particularly on how such age-specific policies may elevate ageist stereotypes in news media reporting. This is an important area of inquiry because public policies have an outsized and a direct impact on society.

The paucity of research could be due to several reasons. First, the link between aging policy and Societal Age Stereotypes (SAS) is tenuous—it is challenging to distill the impact of policy in a competing field of antecedents, such as demographics. Second, the impact of aging policies on societal perceptions may be relatively muted because it targets older adults (and their caregivers), and the wider population may not be aware or interested. Third, amidst the rapid news cycles, public communications of aging policies hardly take a hold on public consciousness to make a discernible difference to societal perceptions. Fourth, a lack of data sets to dynamically track societal perceptions before and after the policy implementation. While surveys are sometimes conducted, they are typically cross-sectional, ad hoc, and post hoc. Because of these Gordian knots, few studies have attempted to analyze the impact of aging policy on SAS.

Against this background, we identified the following guiding principles to select our policy platform. First, the agenda setting for the aging policy must be done at a significant national platform for the policy to gain prominence and media attention (McCombs, 2005). Second, there should be a visible implementation of the aging policy linked to sustained media and public engagement. Third, availability of a dynamic data set that tracks societal perceptions/stereotypes before and after the policy implementation.

Our global policy search identified Asia as a focus region. Demographically, Asia consists of societies that are super-aged (e.g., Japan) and rapidly aging (e.g., Hong Kong, South Korea, and Singapore)—prompting their respective governments to act decisively on the policy front (The Gerontological Society of America [GSA], 2018; United Nations, 2020). Within Asia, our search identified three countries with significant aging policies. Japan, a super-aged society where 25% of the population are aged 65 years and older (United Nations, 2020), implemented a comprehensive Long-Term Care Insurance in 2000—one of the most generous schemes that funds the spectrum of aged care including assistance with grocery shopping (Pang & Arivalagan, 2020). South Korea, an aged society that will reach super-aged status by 2024, implemented the Basic Old-Age Pension Program and Long-Term Care Insurance, though scholars have criticized the insufficiency of these policies to raise the well-being of older adults (Lee & Wolf, 2014).

Singapore, one of the most rapidly aging countries (United Nations, 2020), reached a demographic milestone in 2018, where the number of individuals aged 65 years and older equaled that of youths 15 years and younger. In addition, projections show that disability prevalence will grow by fivefold in 40 years (Ng et al., 2020). The GSA featured Singapore for her strong policy responses in the 2018 Longevity Economics report. Survey studies reveal that seven in 10 older adults reported enjoying a good quality of life (Mathews & Straughan, 2014); however, a minority espoused ageist views that associate old age with physical and mental decline and greater financial dependence (Soin, 2018). While Singapore does not have antiage-discrimination laws, employers could be fined for dismissing older adults on the basis of age (Ministry of Manpower Singapore, 2020). Singapore positions itself as a living laboratory to test out interventions in towns with a higher proportion of older adults such as accessibility schemes that grant older pedestrians extended time to cross busy roads and public housing improvement programs such as the Essential, Optional, and Enhancement for Active Seniors that add nonslip flooring and grab bars in bathrooms to reduce fall risks (Housing and Development Board Singapore, 2021). Despite the good quality of life, espoused by older adults in Singapore, and many ongoing pilots to increase their well-being, Singapore formulated her most ambitious policy—the Pioneer Generation Policy—that exceeded the aforementioned guiding principles. We provide specific reasons for selecting this policy.

First, Singapore’s Pioneer Generation Policy’s agenda setting was done in August 2013 by the Prime Minister of Singapore at his prominent annual address, the National Day Rally Speech (Prime Minister’s Office Singapore, 2013)—akin to the State of the Union Address, or the Speech from the Throne in the United Kingdom. The Prime Minister devoted this 3-h speech to introduce the Pioneer Generation Policy—presented publicly as the Pioneer Generation Package (PGP)—a dedicated policy that honored older adults 65 years and older for their pioneering contributions (Prime Minister’s Office Singapore, 2013).

Second, following the Aging Policy Agenda Setting, the ensuing policy implementation in February 2014 achieved sustained and especially high visibility. A special $9 billion (US$7 billion) fund, making up 15% of the total budget that year (Government of Singapore, 2015, Government of Singapore, 2020), was set up and managed by a new federal agency, the Pioneer Generation Office. A PGP card was provided to all 450,000 citizens who were 65 years and older and was accepted at all public health care institutions to significantly subsidize outpatient costs for specialist clinics including chronic care, dentistry, ophthalmology, and medication from pharmacies (Lim, 2017). The fiscal scale of the PGP was met only by the unparalleled efforts in public communications and outreach. The Pioneer Generation Ambassador Program was set up, where volunteers knocked on almost 400,000 doors to explain the package and offer personalized counseling to older adults and their caregivers. The policy communication efforts included numerous community engagements and videos publicizing the PGP through love stories, retro dances, karaoke, and even fortune-telling (Lee & Yong, 2016). A representative survey found that over 95% of older adults were aware of the PGP and almost 90% knew of the subsidy details for medical costs (Lai, 2016)—indicating the success of the agenda-setting exercise (McCombs, 2005). In contrast, the policies in Japan and South Korea were not publicized at such a gargantuan scale, and some stakeholders were critical of their efficacy, especially in South Korea (Lee & Wolf, 2014).

Third, the availability of a massive database of 10 billion words from over 7,000 online newspapers and magazines, culminating in 30 million articles—provided the appropriate data set to dynamically track annual societal perceptions from before (2010–2013) to after the PGP implementation (2014–2017). On the other hand, there were no known data sets in Japan and South Korea that could measure societal narratives dynamically and with such granularity. Taken together, Singapore provides a compelling microcosm to test the important link between Aging Policy Agenda Setting and SAS.

This study is significant in several ways. Conceptually, it contributes to an underresearched body of knowledge on the impact of policy on societal perceptions—especially, ageism—that have eluded researchers due to several challenges on the policy and data fronts. Practically, the study provides policymakers with a glimpse into how aging policy influences SAS—specifically, it provides a framework to implement aging policies without ageism as an (unintended) consequence.

## Synthesis of Literature on the Impact of Aging Policies on SAS

We synthesize the extant literature to formulate our hypotheses. Fiscally, the federal government is projected to spend over two thirds of its budget on older adults aged 65 and older over the next decade, particularly on Social Security and Medicare policies (Super, 2020). A wide range of other old-age policies in “health insurance; nutritional, legal, supportive, and leisure services; housing; home repair; energy assistance; transportation; help in getting jobs; protection against being fired from jobs; public insurance for employer-sponsored pensions; special mental health programs” (Binstock, 2005, p. 76) also exist to provide aid to older individuals.

The success of these social policies, however, hinges on complex and interacting factors. Overall, while older adults have generally been concluded to have benefitted from these policies, they facilitated negative stereotypes of frailty and dependence (Binstock, 2005). Early work in evaluating older adult policies was similarly concerned with the double-edged sword of having geriatric needs met and their vulnerability to stereotyping (Tropman & McClure, 1978). The yardsticks of successful policy are nuanced, with the possibility of unintended consequences arising beyond the expected scope of the policy, despite best interests (Oliver et al., 2019). For instance, policies that favor older workers via affirmative action may ironically increase negative perceptions toward them (Iweins et al., 2012), and without careful consideration, policies that are meant to serve the interests of older adults may further foster ageist stereotypes (Bugental & Hehman, 2007). Poorly designed elder-specific policies may also unwittingly reinforce stereotypes that older adults are incapable of continued productivity in their old age (Levy & Banaji, 2002). Furthermore, this negative effect compounds over time, as studies suggest that internalizing negative age stereotypes about older adults in one’s youth predisposes individuals to oppose an increase in government spending on welfare programs for older adults (Levy & Schlesinger, 2005).

To synthesize the above literature, we observe that various forms of sociocultural and media paint increasingly negative age stereotypes; mitigated by strong efforts on the part of government policies and activist groups promoting the interests of older adults—though with the possibility of unintended negative outcomes. This article is particularly interested in this dimension: Whether the introduction of huge sums of pro-older-adult government policy may positively affect age stereotypes or succumb to unintentional negative stereotyping, against a cultural backdrop of increasingly negative stereotypes (Lakra et al., 2012; Ng, 2021a; Ng & Chow, 2020; Ng & Indran, 2021a; Ng & Levy, 2018; Ng & Rayner, 2010; Ng et al., 2016; Sima et al., 2013). This conceptual angle provides a potential contribution to assessing the impact of aging policies on SAS. In this vein—and segueing into this article’s main focus—we identified Singapore’s Pioneer Generation Policy as a unique microcosm, which met our policy and data criteria, to investigate the policy–perception relationship.

We test three hypotheses. Hypothesis 1 states that Aging Policy Agenda Setting, as exemplified by Singapore’s Pioneer Generation Policy launched by the Prime Minister in 2014, will be linked to more negative SAS. This is contrary to popular belief as the many public announcements contained highly positive descriptors of older adults (e.g., “special,” “care,” “grateful,” “recognized,” “achieved”), influencing news outlets to frame older adults positively. However, our Hypothesis 1 followed the extant literature that emphasized the unintended consequences of aging policies. Aging Policy Agenda Setting is defined as the PGP’s launch, implementation, and ensuing media coverage from 2014. SAS are defined as societal sentiments of older adults (Ng, 2018; Ng et al., 2015, 2020). Measurement details are provided in the following section.

Hypothesis 2, a mediation hypothesis, states that the association between the Aging Policy Agenda Setting and SAS will be mediated by Medicalization of Aging (defined as the association of physical health and medical terminology with older adults; Heidekrueger et al., 2017; Ng, 2021b; Ng & Chow, 2020; Ng et al., 2012, 2021). With the PGP as a policy primarily focused on health care and medical subsidies, the link between older adults and physical ailments may be unwittingly strengthened, promoting the Medicalization of Aging, and consequently, increasing negative age stereotypes. Studies show the increasing societal tendency to associate older adults with physical illnesses and ailments, and as patients instead of individuals with varied and fulfilling life experiences (Binney et al., 1990; Glover & Harman, 2000).

Hypothesis 3, a moderated mediation hypothesis, states that the link between Aging Policy Agenda Setting and the Medicalization of Aging will be moderated by Old-age Support Ratio (ratio of the working-age population aged 20–64 per person aged 65 years and older). Prior studies found that countries with a higher proportion of older adults older than 65 years tended to espouse more negative age stereotypes (Löckenhoff et al., 2009). Given the rapid aging in Singapore, coupled with one of the lowest fertility rates over the past two decades, the decreasing Old-age Support Ratio over the 8-year study period provides a suitable gauge of how demographics would moderate the impact of aging policies on the Medicalization of Aging.

## Method

### Data Set

To test our hypotheses, we used the News on the Web corpus (Davies, 2017), a massive and dynamic corpus consisting of content from 7,000 web-based newspapers and magazines from 20 countries over 10 years. The 10-billion-word database consists of more than 30 million articles with 300,000 new articles added monthly. This data set was conceptualized with grants from the National Science Foundation and the National Endowment for the Humanities to study contemporary English language usage in countries where English is widely used, spanning six regions: North America (America, Canada); Europe (Ireland, the United Kingdom); Oceania (Australia, New Zealand); Asia (Bangladesh, Hong Kong, India, Malaysia, Pakistan, Philippines, Singapore, Sri Lanka); Africa (Ghana, Kenya, Nigeria, South Africa, Tanzania); The Caribbean (Jamaica). Within our study’s timeframe (2010–2017), the Singapore corpus consists of 188 million words. According to Cultivation Theory, the large representation of online media in the corpus is appropriate in reflecting societal perceptions encoded in the country (Gerbner et al., 1994).

### Measurement of SAS and Medicalization of Aging

We extracted all texts within the corpus that contained the target words related to older adults. In Singapore, “elderly” is the most commonly used noun to refer to an older adult and it is used as a neutral noun—though we acknowledge its derogatory connotation and the movement against it (Berridge & Martinson, 2017). From a data perspective, “elderly” was the most frequently used noun (45.81 per million), compared to “older adult” (0.72 per million), and was used as the target noun.

Next, we preprocessed the 188-million-word data set by excluding prepositions, conjunctions, and other “stop words” (e.g., *an*, *the*) found within the text. We then shortlisted all collocates of the target word (i.e., words that co-occurred most closely and frequently with it) for each year. Collocates were shortlisted based on the following criteria: (a) Lexical Proximity: The collocate appears within six words before or after the target word. Articles such as “the,” “a” were not included in the six-word lexical span. If the target noun was the first word of a sentence, the collocates from the prior sentence were excluded. (b) Relevant context: The collocate was used to refer specifically to an old person (checked by two raters). (c) Mutual Information Score of 1.5 and above: The collocate has a stronger association with the target word compared to other words in the corpus, indicating semantic bonding (Church & Hanks, 1990). This method, known as Psychomics, is an application of concordance analysis—used in computational linguistics to analyze language evolution—to identify changes in stereotypes, sentiments, and narratives in recent studies (Ng & Indran, 2021b, 2021c, 2021d; Ng & Lim, 2020; Ng & Tan, 2021a; Ng et al., 2015). This rigorous process culminated in 5,460 collocates across 8 years.

Thereafter, each collocate, which met our study criteria, was rated on a scale from 1 (*very negative*) to 5 (*very positive*) by two independent raters—a method found to be valid and reliable to appraise age-stereotype-associated words (Cronbach’s alpha: 0.958, 95% confidence interval [CI]: 0.792–0.992; Levy & Langer, 1994). For instance, *abuse*, *debilitating*, *suffering* were rated 1 (*very negative*); *employee*, *occupational*, *shoe* were rated 3 (*neutral*); *empowering*, *hero*, *venerable* were rated 5 (*very positive*). Mean ratings per year were calculated to produce an SAS score, per year, across the 8 years of analysis (2010–2017) to test Hypothesis 1.

To address Hypothesis 2—consistent with previous studies (Ng & Chow, 2020; Ng & Tan, 2021b)—we obtained an annual “Medicalization of Aging” score by rating all 5,460 collocates on a binary scale (1 = yes, 0 = no) based on the question: “Does this word describe or related to the physical health of a person?” Collocates such as *sick*, *dementia*, *diabetic*, *comorbidities*, *illness*, *rheumatism*—among other medical-related terms about treatment, medication, and chronic illnesses—were examples of words that were rated 1. “Old-age Support Ratio” per year from 2010 to 2017 (the study period) was obtained from Singapore’s Department of Statistics (Government of Singapore, 2019). All data preprocessing, text analytics, and statistical analyses were conducted in Python 3.7 and OriginPro 2019b.

### Analytic Strategy

Hypothesis 1—states that the Aging Policy Agenda Setting in 2014 will be linked to more negative SAS—is tested via a trend analysis. Hypothesis 2 (Mediation Hypothesis) states that the association between the Aging Policy Agenda Setting and SAS will be mediated by Medicalization of Aging, and Hypothesis 3 (Moderated Mediation Hypothesis) states that the link between Aging Policy Agenda Setting and the Medicalization of Aging will be moderated by Old-age Support Ratio. Both Hypotheses 2 and 3 will be tested by Edwards and Lambert (2007)’s General Path Analytic Framework that extends well-known approaches (Baron & Kenny, 1986) with the following benefits: First, the framework provides the conceptual flexibilities of a moderator variable exerting an effect on one or more paths of a basic mediation model. Second, the combination of moderation and mediation does not produce a single path model (the norm in previous approaches) but yields a variety of models that allow for direct, indirect, and total effects at a given level of the moderator variable.

## Results

### SAS Over 8 Years From Pre- to Postaging Policy Implementation

**Figure 1.**
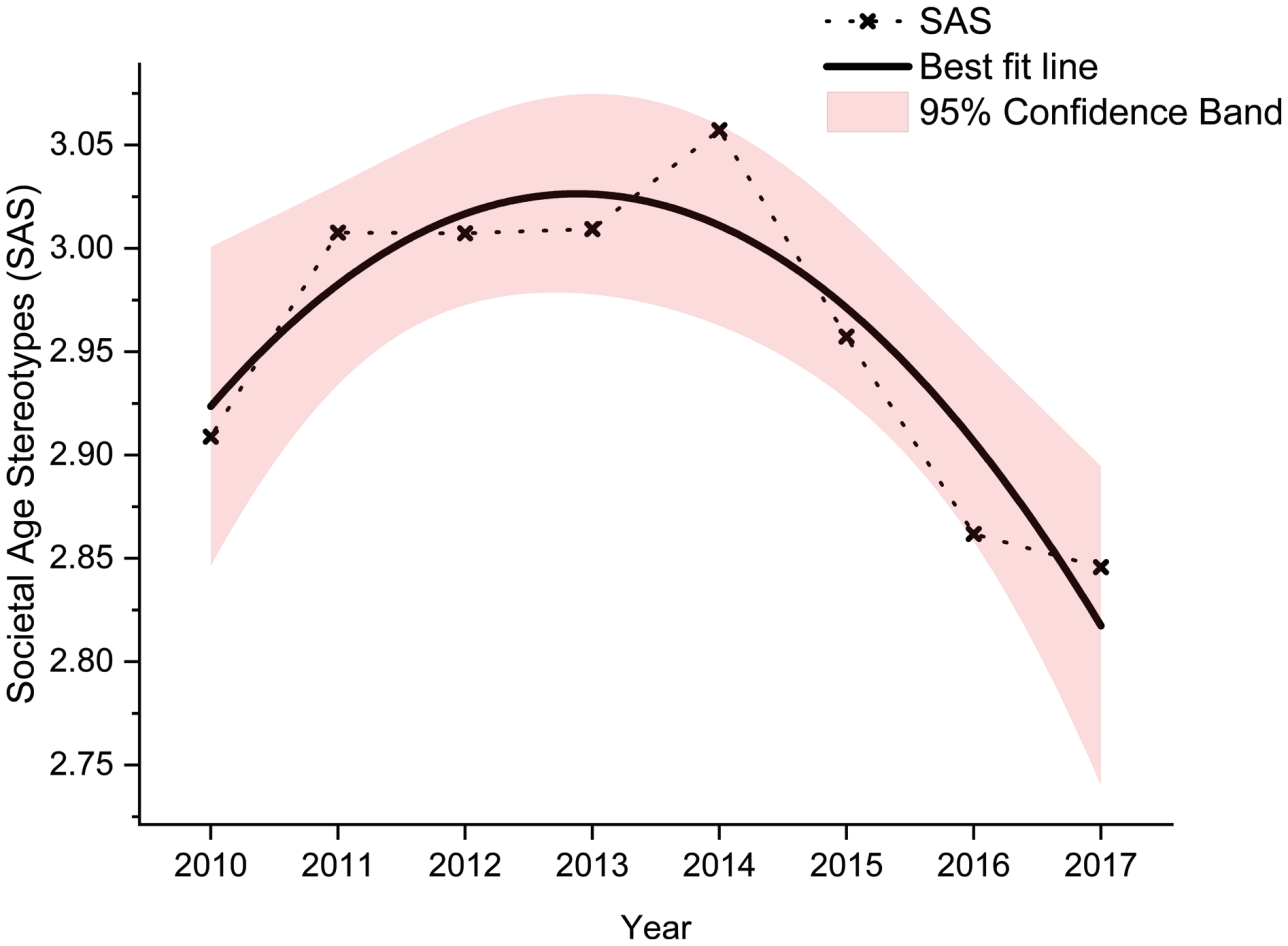
Societal Age Stereotypes scores from 2010 to 2017 followed a quadratic trend. Prior to the Aging Policy Agenda Setting from 2010 to 2014, stereotypes were trending positive; after 2014, it trended downward to become more negative.

Overall, SAS followed a quadratic trend (β = −0.0123, *p* = .00645): Prior to the Aging Policy Agenda Setting from 2010 to 2014, stereotypes were trending positive; after 2014, it trended downward to become more negative—providing support for Hypothesis 1 (Figure 1). The cubic trend did not reach significance (β = 0.001, *p* = .456). Old-age Support Ratio decreased over 30% in the similar 8-year span, from 7.4 in 2010 to 5.1 in 2017—indicating the rapid aging of Singapore in less than one decade.

### Pathway Analysis: Mediation and Moderated Mediation

#### Mediation

**Figure 2.**
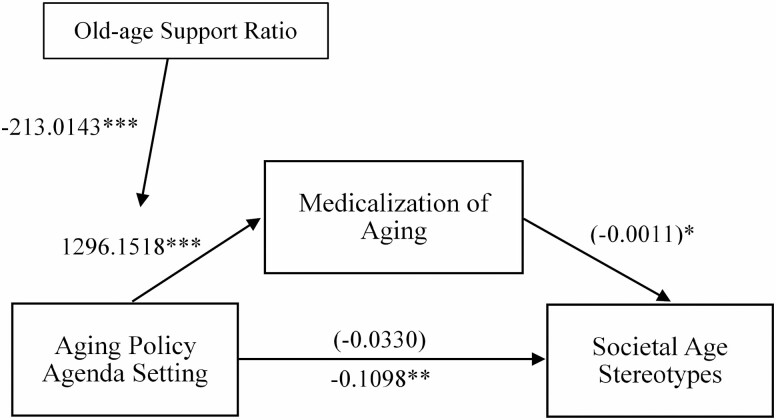
The overall path diagrams for the mediation and moderated mediation models. The values refer to the unstandardized coefficients. Values in parenthesis refer to the respective coefficients of the indirect effect of Medicalization of Aging, after controlling for Aging Policy Agenda Setting. *Note:* *p < 0.1, **p < .05, ***p < .01.

We tested whether Medicalization of Aging mediated the relationship between Aging Policy Agenda Setting and SAS. The results indicated a significant total direct effect (without mediator) of Aging Policy Agenda Setting on SAS, β = −0.1098, *t* = −2.671, *p* = .037, 95% CI = −0.210 to −0.009, *R*^2^ = 0.543; a significant direct effect (with mediator), β = 135.85, *t* = 4.477, *p* = .004, 95% CI = 61.596–201.109, *R*^2^ = 0.729; and a significant indirect effect through Medicalization of Aging, β = −0.0011, 95% CI = −0.002 to 0.000, *p* = .09, after controlling for Aging Policy Agenda Setting that did not reach significance (β = 0.0330, 95% CI = −0.159 to 0.225, *p* = .677)—indicating full mediation (Figure 2).

#### Moderated mediation

**Figure 3.**
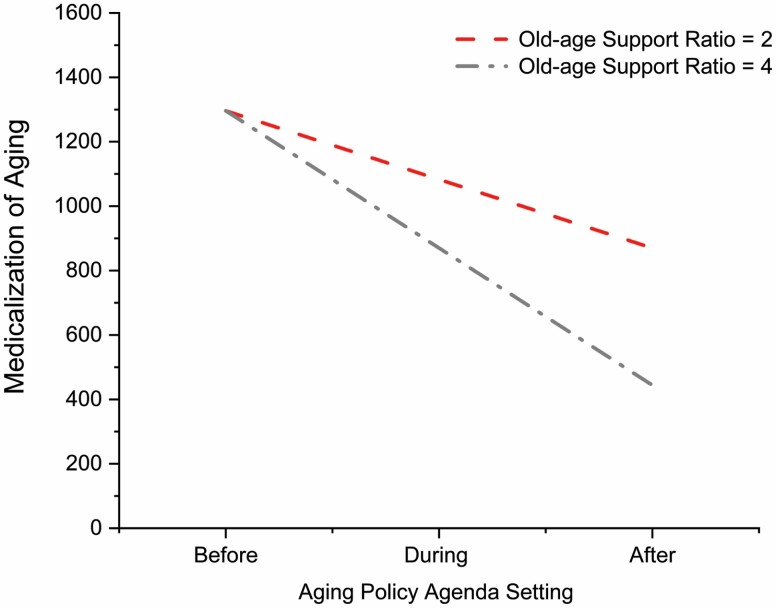
The impact of Aging Policy Agenda Setting on Medicalization of Aging is stronger at a lower Old-age Support Ratio as shown in the moderation analysis. This suggests that as a society ages—as indicated by the decreasing number of economically active adults supporting an increasing number of older adults who are materially dependent—the impact of aging policies on the Medicalization of Aging becomes more profound.

As hypothesized, the interaction between Aging Policy Agenda Setting and Old-age Support Ratio reached significance, *β* = −213.01, *p* = .005, indicating that the indirect effect of Aging Policy Agenda Setting on SAS through Medicalization of Aging was moderated by Old-age Support Ratio—providing support for Hypothesis 3 (Figure 2). A simple moderation analysis was also conducted to explore the interaction effect (Aging Policy Agenda Setting × Old-age Support Ratio) on Medicalization of Aging. We found that the impact of Aging Policy Agenda Setting on the Medicalization of Aging is stronger at a lower Old-age Support Ratio (Figure 3). In essence, when a society ages—as indicated by the decreasing number of economically active adults supporting an increasing number of older adults who are materially dependent—the impact of aging policies on the Medicalization of Aging becomes more profound.

## Discussion

Our study is among the few that analyzed the impact of aging policies on SAS, measured annually across 8 years, from pre- to postpolicy implementation—providing an unprecedented (longitudinal) glimpse into the unintended consequences of aging policies in promoting ageism. While previous studies have explored the policy–ageism link, this is one of the first to elucidate potential pathways, laying the groundwork for policymakers to implement aging policies without a corresponding increase in societal ageism.

Consistent with previous studies, we found support for increased ageism following a major agenda setting for an aging policy. Our pathway analyses showed that the aging policy—in the form of Singapore’s Pioneer Generation Policy that provided close-to-full subsidies for all outpatient costs and monthly payouts for disability—was linked to increased Medicalization of Aging and a subsequent decline in SAS, where perceptions of older adults became more negative. Importantly, the impact of aging policy on increased Medicalization of Aging is more profound at lower Old-age Support Ratios.

Our findings are significant in several ways. First, we elucidated the enabling factors for aging policy to affect SAS. These guiding principles provide scholars with a conceptual framework for aging policy analysis, novel data platforms, and analytical strategies—contributing to the growing body of policy analysis that mainly employed interviews and surveys (Levy & Schlesinger, 2005). The enabling factors for aging policy analysis are as follows: (a) Agenda Setting for the aging policy performed at significant national/federal platforms to gain prominence and media attention (McCombs, 2005); (b) visible and sustained implementation; (c) availability of dynamic data platforms to track societal perceptions longitudinally from pre- to postpolicy agenda setting.

Second, the policy antecedence of ageism is understudied. Several studies have documented the paradox of aging policies, where the policy intention to increase the well-being of older adults (financially or health) is ironically linked to ageism; however, no known studies have investigated the policy–ageism pathways. Ageism is an important topic given its insidious consequences: Ageism is linked to $63 billion per year of health care cost (Levy et al., 2020), increased cardiovascular risk (Levy et al., 2009), and premature mortality (Levy et al., 2002). Specifically, we found that aging policies increased ageism via the Medicalization of Aging, and this link is stronger at lower Old-age Support Ratios.

The Pioneer Generation Policy’s focus on medical subsidies may have unwittingly strengthened the relationship between older adults and physical ailments. Significant emphasis in public communications was about the increased incidence of chronic conditions in older adults, increasing costs of chronic care, and the policy’s benefit to cover medical costs for life (Lim, 2017). For instance, older adults will receive close-to-full subsidies for outpatient visits in primary, specialized, and dental care and medications; annual cash checks of $1,200 (US$920) provided to those with moderate to severe disabilities (Lai, 2016). Unfortunately, these conditions set up a stronger tendency for older adults to be increasingly associated with illness, disability, and death. Consistent with previous studies, well-intentioned public policies such as the PGP may have unintentionally driven long-term ageism due to its increased attention on mortality and sickness.

These findings contribute to the evaluation of wide-reaching age policies, and in determining whether age stereotypes were inadvertently reinforced after its implementation. With the large amount of institutional funding that such policies demand, it is prudent to consider these unintended consequences. Our findings suggest that, against a backdrop of increasingly negative portrayals of older adults in various forms of media (Ng & Chow, 2020), well-intentioned government policies may ironically backfire and exacerbate this trend. The policy has unintentionally strengthened the link between older adults and medicalization—illness and death—particularly in newspapers, magazines, and blogs that may result in greater negative age stereotyping. Should these trends persist and become entrenched in public consciousness, such negativity will engender long-lasting psychological and physical impact on the perceptions of older adults.

Third, and practically, there is an urgent need for governments of rapidly aging countries to intervene, given the significant moderated mediation effect where the impact of aging policies on the Medicalization of Aging is stronger within more aged societies. From an intervention standpoint, there are innovations at the individual (Lytle et al., 2020) and societal levels (American Association of Retired Persons (AARP), 2020), but few studies focus on policymakers (Ng & Lim, 2020; Ng & Wong, 2021). The prevalence of ageism in public policy is due to a lack of awareness. Ageism is the least acknowledged form of prejudice, compared to racism and sexism. Future initiatives could focus on policymakers by increasing their awareness of ageism within public policy and the unintended impact of ageism from health policies. Practically, our study provides a framework to analyze how aging policies could promote ageism via the Medicalization of Aging. While the latter might be inevitable, though unintended, existing policy communications could include an additional component to highlight positive stories of resilience and wellness to counteract the negativity. In addition, most aging policies frame older adults based on their age, labeling them as “senior citizens” or “old people”—such age-based framing is linked to increased ageism (Ng & Indran, 2021b, 2021c; Ng et al., 2021a; Ng et al., 2021b; Ng et al., 2015). Though policies are typically age-based, policymakers could promote role-based framing, emphasizing older adults’ familial (e.g., grandparenting) and professional roles to ameliorate ageism (Ng & Indran, 2021b). Future studies could explore the efficacy of interventions targeted at policymakers.

Our study suffers from a significant data limitation as our dynamic database did not include social media where disparaging phrases like “ok boomer” have intensified in recent years. The diversity of social media usage across multiple platforms makes data collation challenging, and most platforms such as Facebook are closed to public access—they have also become increasingly monetized, selling selected data sets that may not be representative. Nevertheless, a future iteration of the study should include social media, for comparative analysis across cultures (Ng, 2013a, 2013b, 2013c, 2013d) to facilitate advocacy campaigns.

In conclusion, the impact of aging policies on increasing societal ageism cannot be overlooked. This is one of the first known studies to analyze the trajectory of SAS across an 8-year longitudinal timeframe from pre- to postagenda setting, and presented a plausible pathway via Medicalization of Aging, and moderated by demographics. Though it is laudable that major strides have been achieved for more policies, our study shed light on the unintentional consequences. We hope to have provided a framework for policymakers and scholars to ameliorate the unintended consequences of aging policies on societal ageism—if unaddressed, ageism will exert a significant toll on the well-being of older adults, even if initial policies are well-intentioned.

## Data Availability

Data are publicly available at https://www.english-corpora.org.
